# Supplementary material for: CONSERVATION AND DIVERGENCE WITHIN THE ARABIDOPSIS TPL/TPR COREPRESSOR FAMILY
Source: bioRxiv. 2026 Jun 5:2026.06.02.729393. Preprint. [Version 1] doi: 10.64898/2026.06.02.729393 (PMC13251949; doi:10.64898/2026.06.02.729393)
Supplement: Supplement 1 [file NIHPP2026.06.02.729393v1-supplement-1.pdf]

bioRxiv preprint doi: https://doi.org/10.64898/2026.06.02.729393; this version posted June 6, 2026. The copyright holder for this preprint (which was not certified by peer review) is the author/funder, who has granted bioRxiv a license to display the preprint in perpetuity. It is made available under a CC-BY 4.0 International license.

Figure Supplements

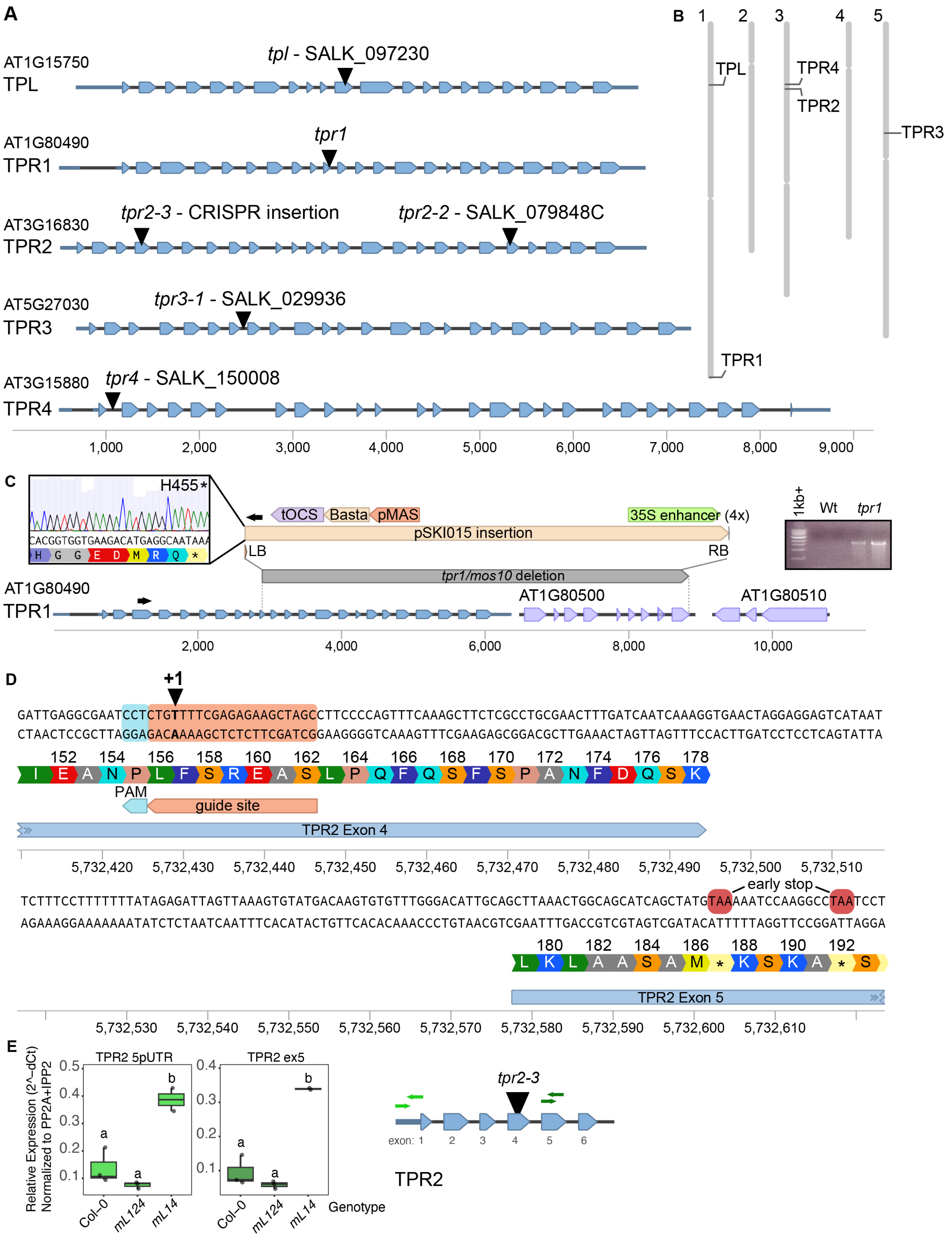

bioRxiv preprint doi: https://doi.org/10.64898/2026.06.02.729393; this version posted June 6, 2026. The copyright holder for this preprint (which was not certified by peer review) is the author/funder, who has granted bioRxiv a license to display the preprint in perpetuity. It is made available under a CC-BY 4.0 International license.

Figure 1 - figure supplement 1. TPX gene models, mutant alleles, and CRISPR design. (A) Gene models for *TPL* (SALK_097230), *TPR1* (T-DNA induced deletion), *TPR2* (*tpr2-3* CRISPR insertion; *tpr2-2* SALK_079848C), *TPR3* (SALK_029936), *TPR4* (SALK_150008). Scale in bp. **(B)** Chromosomal positions of all five genes. **(C)** Detailed sequence of the *tpr1-1/mos10* allele showing pSKI015 insertion, orientation, left and right borders. Electrophoresis gel of targeted amplification of the *tpr1* allele, with associated Sanger sequencing shown in the inset: the allele induces an early stop at H455. **(D)** Detailed sequence of the *tpr2-3* CRISPR allele showing guide site at the Exon 4/5 junction, amino acid positions 152–192, and premature stop codons introduced within the CRA domain. **(E)** qPCR with four primer sets, upstream and downstream of the CRISPR insertion site, confirms reduction of *TPR2* expression in *tpr2-3*.

bioRxiv preprint doi: https://doi.org/10.64898/2026.06.02.729393; this version posted June 6, 2026. The copyright holder for this preprint (which was not certified by peer review) is the author/funder, who has granted bioRxiv a license to display the preprint in perpetuity. It is made available under a CC-BY 4.0 International license.

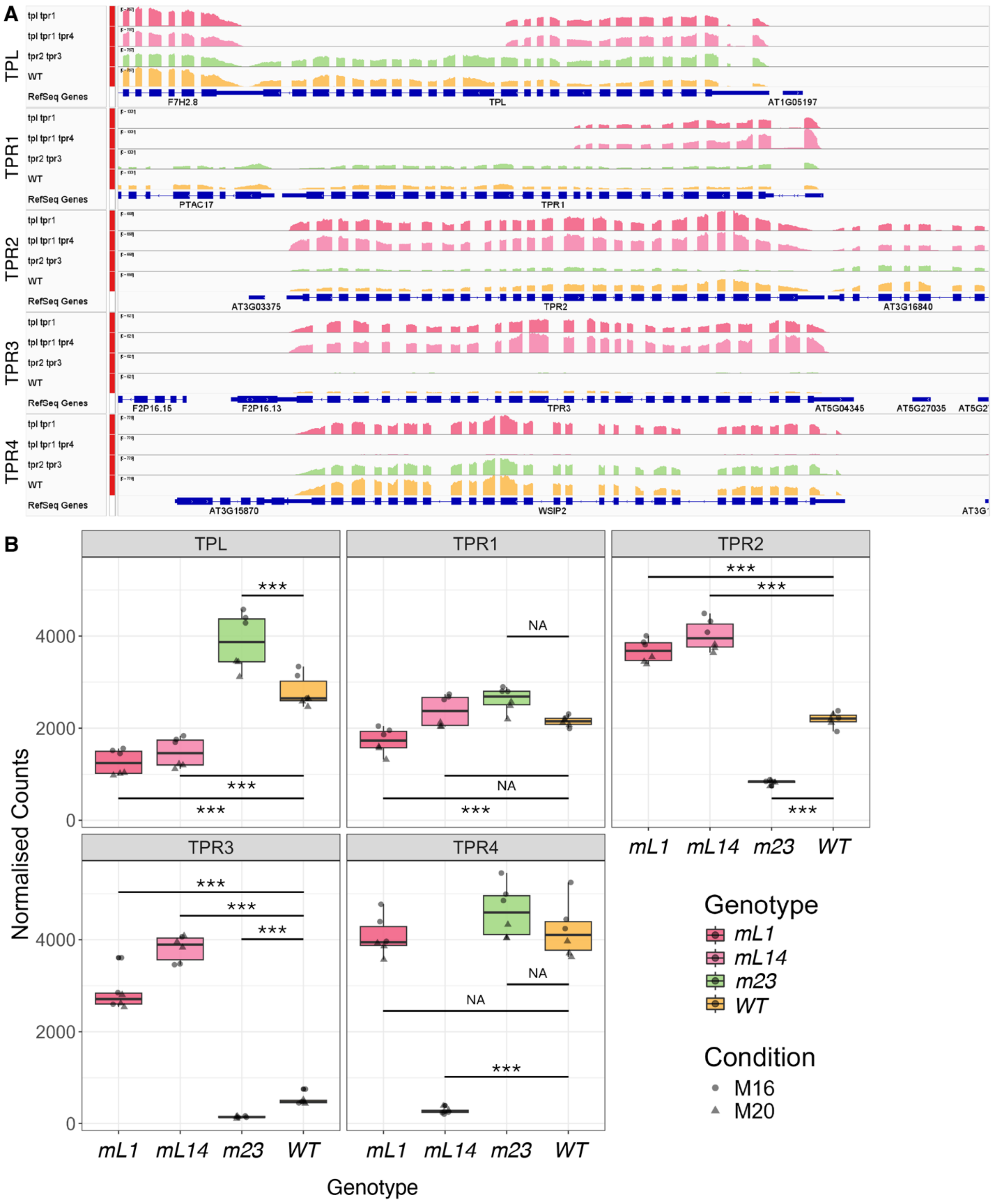

Figure 2 - figure supplement 1. Molecular characterization of the impact of TPX mutants on family member expression by RNA-Seq. (A) Sequence tracks for each of the 5 TPX family members (TPL, TPR1-4) analyzed in multiple mutant backgrounds. These results confirm an absence of full-length mRNA for each of the T-DNA based

bioRxiv preprint doi: https://doi.org/10.64898/2026.06.02.729393; this version posted June 6, 2026. The copyright holder for this preprint (which was not certified by peer review) is the author/funder, who has granted bioRxiv a license to display the preprint in perpetuity. It is made available under a CC-BY 4.0 International license.

mutants defined in Figure 1 - figure supplement 1. The *tpr1* allele contains a deletion (see Figure 1 – figure supplement 1), and this results in no detectable RNA downstream of the deletion site detectable by RNA-seq. RNA levels from the residual locus in *tpr1* mutants are unchanged in aggregate compared to wild type, which suggests upregulation of the transcript in the absence of full length TPR1 protein. **(B)** Quantification of total normalized counts over each gene body plotted as boxplots. *** indicates a significant difference between genotypes ($p<0.01$). NA – no significant difference. These results support the cross-family regulation observed by RT-qPCR in Figure 1.

bioRxiv preprint doi: https://doi.org/10.64898/2026.06.02.729393; this version posted June 6, 2026. The copyright holder for this preprint (which was not certified by peer review) is the author/funder, who has granted bioRxiv a license to display the preprint in perpetuity. It is made available under a CC-BY 4.0 International license.

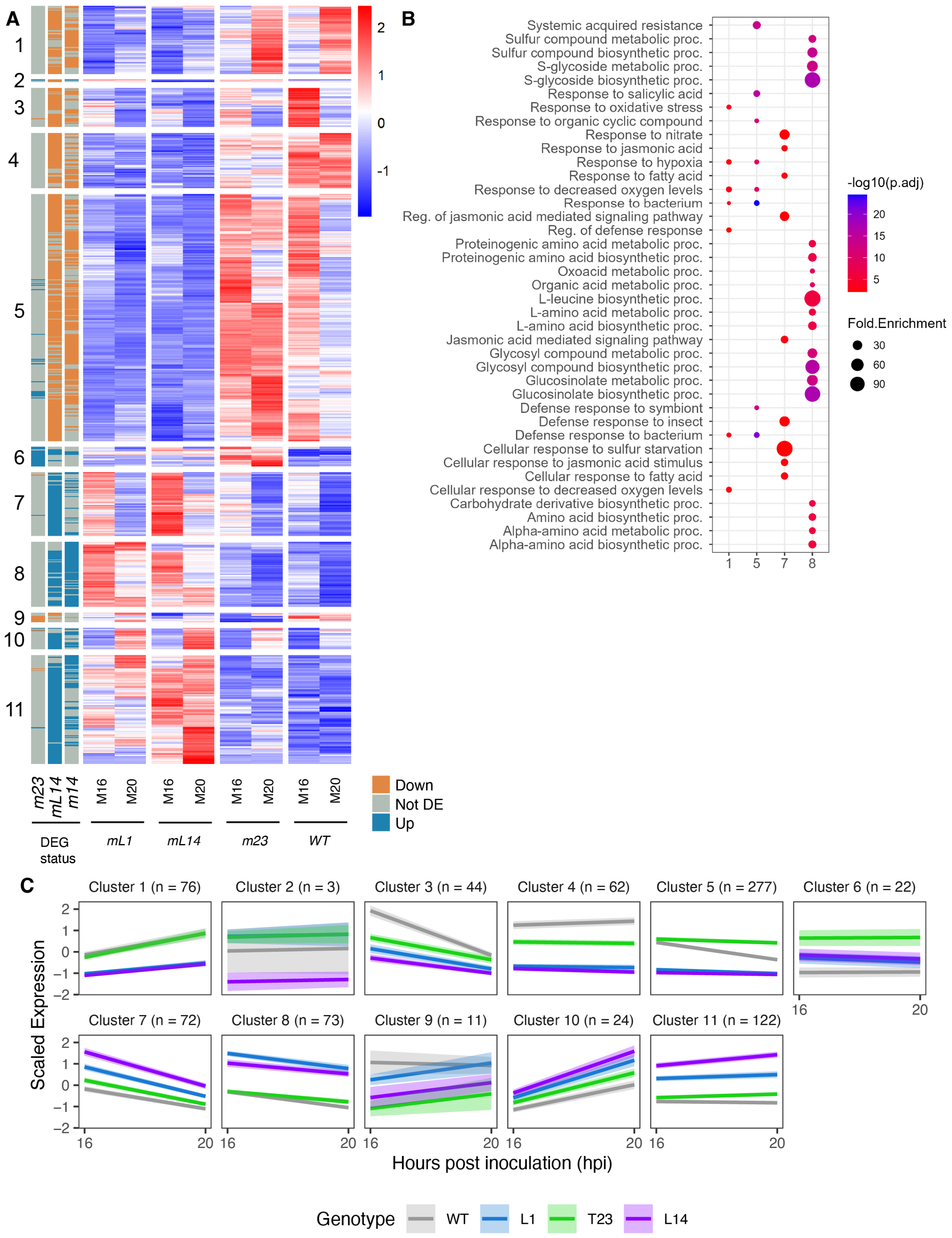

bioRxiv preprint doi: https://doi.org/10.64898/2026.06.02.729393; this version posted June 6, 2026. The copyright holder for this preprint (which was not certified by peer review) is the author/funder, who has granted bioRxiv a license to display the preprint in perpetuity. It is made available under a CC-BY 4.0 International license.

Figure 2 - figure supplement 2. TPX family multiple mutants demonstrate a semi-overlapping impact on the leaf transcriptome.

(A) Heatmap showing row-scaled (z-score) normalized expression of differentially expressed genes (DEGs) identified in mL1, mL14, and m23 relative to wild type under mock conditions (padj < 0.01, |log2FC| ≥ 0.5). Columns represent mean normalized counts ordered by genotype (WT, mL1, m23, mL14) at 16 and 20 hours post inoculation (hpi). Row annotations indicate whether a gene is significantly differentially expressed in *mL1, mL14,* and *m23* compared to WT (Downregulated is blue; Upregulated is orange, not differentially expressed is grey). **(B)** Gene Ontology Biological Process enrichment of DEGs from each transcriptional module identified in (A). **(C)** Mean scaled expression profiles (z-score) of genes within each of the 11 transcriptional modules defined by hierarchical clustering of DEGs in *mL1, mL14, and m23* relative to WT (see A), across 16 and 20 hpi under mock conditions. Lines are colored by genotype (WT, gray; mL1, blue; m23, green; mL14, purple) and shaded ribbons indicate 95% confidence intervals.

bioRxiv preprint doi: https://doi.org/10.64898/2026.06.02.729393; this version posted June 6, 2026. The copyright holder for this preprint (which was not certified by peer review) is the author/funder, who has granted bioRxiv a license to display the preprint in perpetuity. It is made available under a CC-BY 4.0 International license.

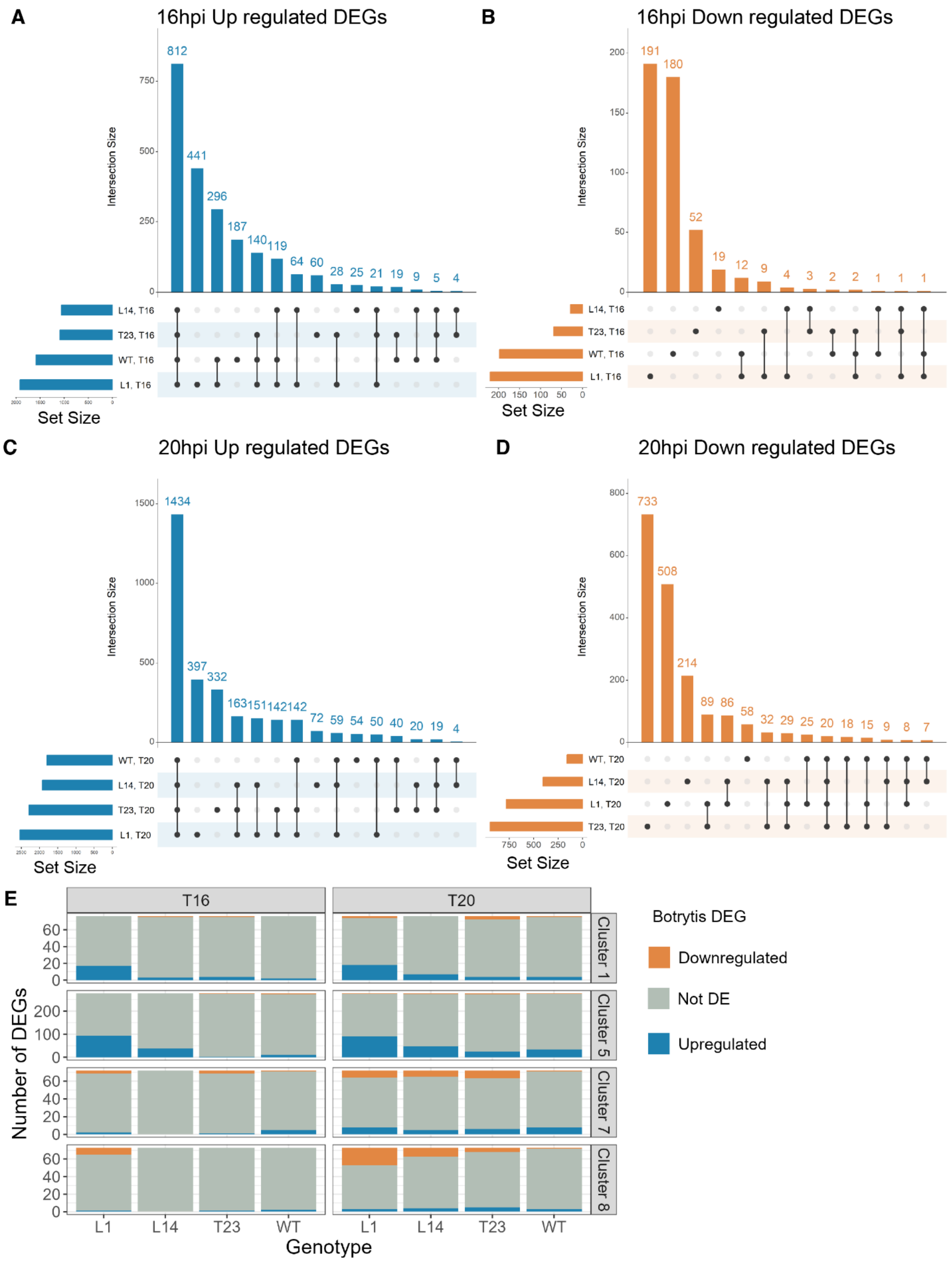

bioRxiv preprint doi: https://doi.org/10.64898/2026.06.02.729393; this version posted June 6, 2026. The copyright holder for this preprint (which was not certified by peer review) is the author/funder, who has granted bioRxiv a license to display the preprint in perpetuity. It is made available under a CC-BY 4.0 International license.

Figure 2 - figure supplement 3. UpSet plots showing overlap of differentially expressed genes in response to *Botrytis cinerea* infection across genotypes. UpSet plots showing the overlap of upregulated **(A, C)** and downregulated **(B, D)** differentially expressed genes (DEGs) identified in response to *B. cinerea* infection relative to mock (padj < 0.01, |log2FC| ≥ 0.5) in wild type (WT), *tpl tpr1* (*mL1*), *tpl tpr1 tpr4* (*mL14*), and *tpr2 tpr3* (*m23*) at 16 hpi **(A, B)** and 20 hpi **(C, D)**. Bar height indicates the number of DEGs unique to or shared between each genotype combination. Set size bars indicate the total number of DEGs per genotype. **(E)** Bar graph showing the genes in four transcriptional modules of DEGs in *tpl tpr1* (*mL1*), *tpl tpr1 tpr4* (*mL14*), and *tpr2 tpr3* (*m23*) mutants compared to wild type (WT) from the mock data (Figure 2 – figure supplement 2) and whether the same genes are differentially expressed in each of the genotypes in response to *B. cinerea* expression at 16 and 20 hours post infection. Orange represents genes that are downregulated upon *B. cinerea* infection, Blue represents genes that are upregulated in response to *B. cinerea* infection, and Grey represents genes that are not significantly differentially expressed in each genotype in response to *B. cinerea* infection.

bioRxiv preprint doi: https://doi.org/10.64898/2026.06.02.729393; this version posted June 6, 2026. The copyright holder for this preprint (which was not certified by peer review) is the author/funder, who has granted bioRxiv a license to display the preprint in perpetuity. It is made available under a CC-BY 4.0 International license.

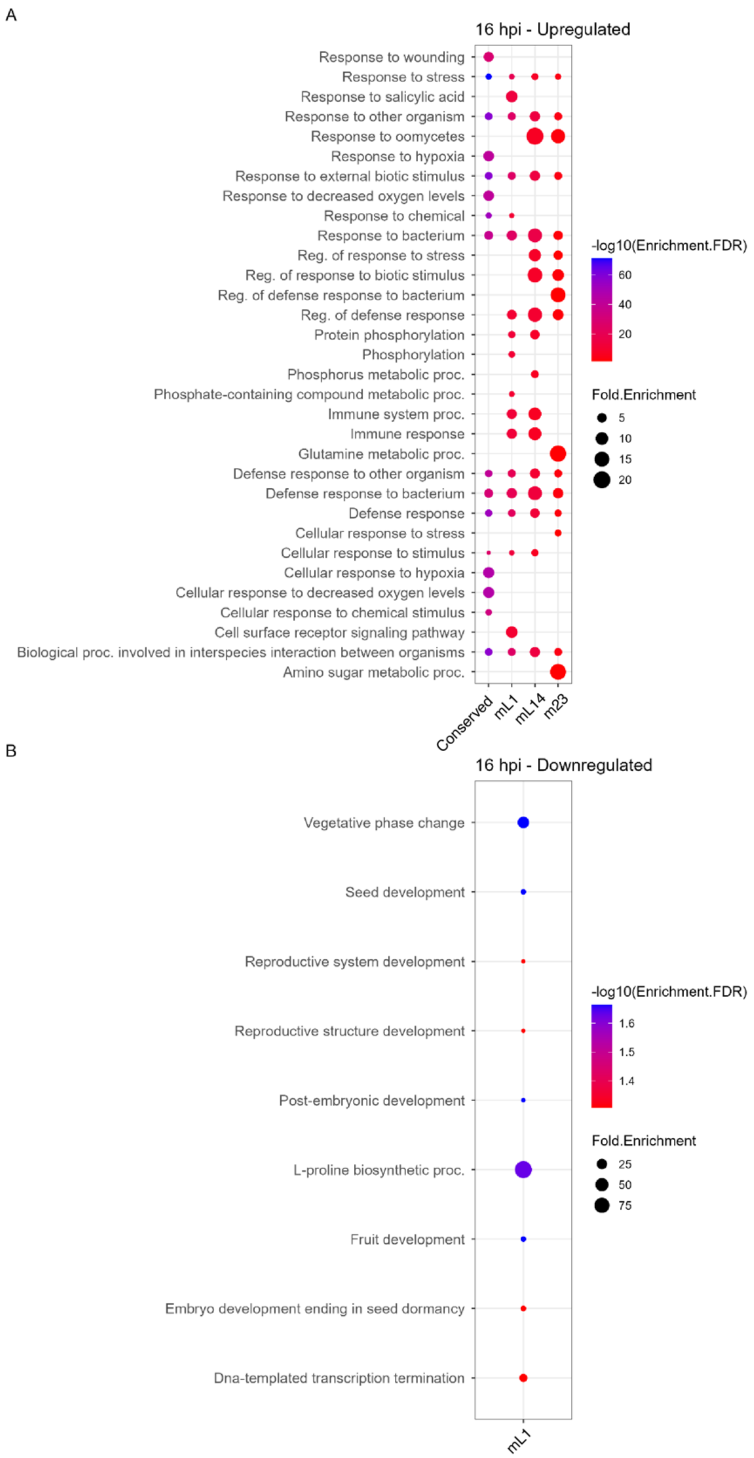

Figure 2 Figure Supplement 4. GO term analysis for conserved and mutant-specific DEGs after *B. cinerea* infection at 16 hpi. **(A)** GO term analysis for conserved (n = 1400), *tpl tpr1* specific (*mL1*, n = 554), *tpl tpr1 tpr4* specific (*mL14*, n = 114), and *tpr2 tpr3* specific (*m23*, n = 113) upregulated DEGs at 16 hpi. **(B)** GO term analysis for conserved (n = 18), *tpl tpr1*specific (*mL1*, n = 205), *tpl tpr1 tpr4* specific

bioRxiv preprint doi: https://doi.org/10.64898/2026.06.02.729393; this version posted June 6, 2026. The copyright holder for this preprint (which was not certified by peer review) is the author/funder, who has granted bioRxiv a license to display the preprint in perpetuity. It is made available under a CC-BY 4.0 International license.

(*mL14*, n = 27), and *tpr2 tpr3* specific (*m23*, n = 65) downregulated DEGs at 16 hpi. Conserved genes were defined as being expressed in WT and at least one of the mutants, in the same direction at the same timepoint; mutant-specific genes were defined as being differentially expressed in the mutant but not in WT, at the same timepoint.

bioRxiv preprint doi: https://doi.org/10.64898/2026.06.02.729393; this version posted June 6, 2026. The copyright holder for this preprint (which was not certified by peer review) is the author/funder, who has granted bioRxiv a license to display the preprint in perpetuity. It is made available under a CC-BY 4.0 International license.

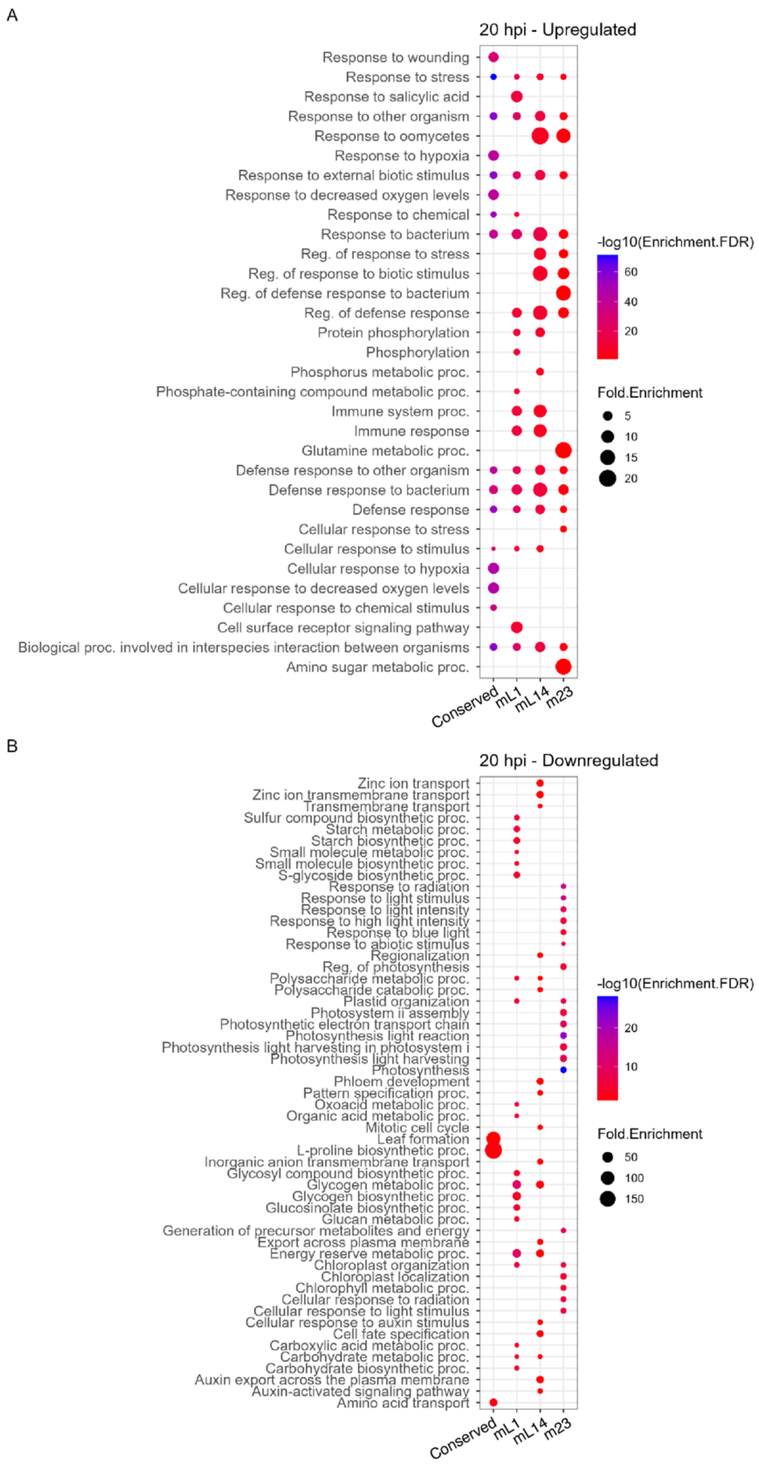

Figure 2 Figure Supplement 5. GO term analysis for conserved and mutant-specific DEGs after *B. cinerea* infection at 20 hpi. (A) GO term analysis for conserved (n = 1748), *tpl tpr1* specific (*mL1*, n = 853), *tpl tpr1 tpr4* specific (*mL14*, n = 406), and *tpr2 tpr3* specific (*m23*, n = 657) upregulated DEGs at 20 hpi. **(B)** GO term analysis for conserved (n = 102), *tpl tpr1*specific (*mL1*, n = 712), *tpl tpr1 tpr4* specific

bioRxiv preprint doi: https://doi.org/10.64898/2026.06.02.729393; this version posted June 6, 2026. The copyright holder for this preprint (which was not certified by peer review) is the author/funder, who has granted bioRxiv a license to display the preprint in perpetuity. It is made available under a CC-BY 4.0 International license.

(*mL14*, n = 361), and *tpr2 tpr3* specific (*m23*, n = 883) downregulated DEGs at 20 hpi. Conserved genes were defined as being expressed in WT and at least one of the mutants, in the same direction at the same timepoint; mutant-specific genes were defined as being differentially expressed in the mutant but not in WT, at the same timepoint.

bioRxiv preprint doi: https://doi.org/10.64898/2026.06.02.729393; this version posted June 6, 2026. The copyright holder for this preprint (which was not certified by peer review) is the author/funder, who has granted bioRxiv a license to display the preprint in perpetuity. It is made available under a CC-BY 4.0 International license.

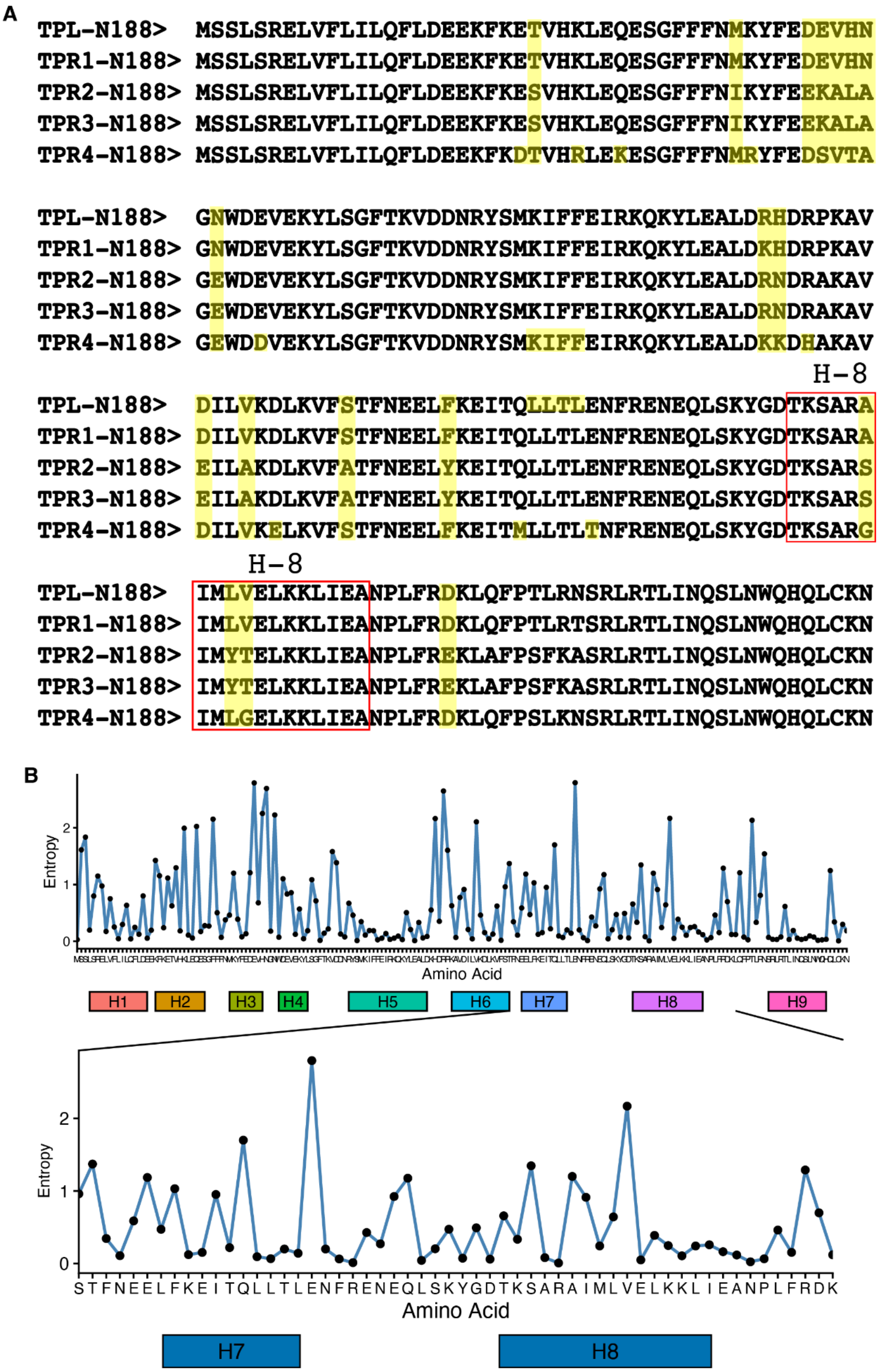

bioRxiv preprint doi: https://doi.org/10.64898/2026.06.02.729393; this version posted June 6, 2026. The copyright holder for this preprint (which was not certified by peer review) is the author/funder, who has granted bioRxiv a license to display the preprint in perpetuity. It is made available under a CC-BY 4.0 International license.

Figure 3 - figure supplement 1. Amino acid sequence alignment and variation in TPX family proteins. (A) Amino acid alignment of TPL and TPR1-4. Alignment performed in MEGA, variant residues are highlighted in yellow. Helix 8 region is denoted with a red box. **(B)** Per-residue sequence entropy along the protein, computed with PoET. Sequence divergence was quantified using PoET (Protein Evolutionary Transformer), an autoregressive, retrieval-augmented protein language model from OpenProtein.AI that models whole protein families as sequences-of-sequences without requiring a multiple sequence alignment. Conditioned on a homologous prompt set drawn from the target family, PoET returns a per-position log-likelihood over the 20 amino acids, which we converted to relative frequencies and summarized as Shannon entropy at each residue. Entropy (y-axis) is plotted against residue position (x-axis) across the full reference sequence. Peaks indicate positions tolerant of substitution and representative of diverged/variable sites ("mutational hotspots"), whereas troughs mark evolutionarily constrained, conserved positions. Analysis was performed on openprotein.ai servers (Truong and Bepler, 2023)

bioRxiv preprint doi: https://doi.org/10.64898/2026.06.02.729393; this version posted June 6, 2026. The copyright holder for this preprint (which was not certified by peer review) is the author/funder, who has granted bioRxiv a license to display the preprint in perpetuity. It is made available under a CC-BY 4.0 International license.

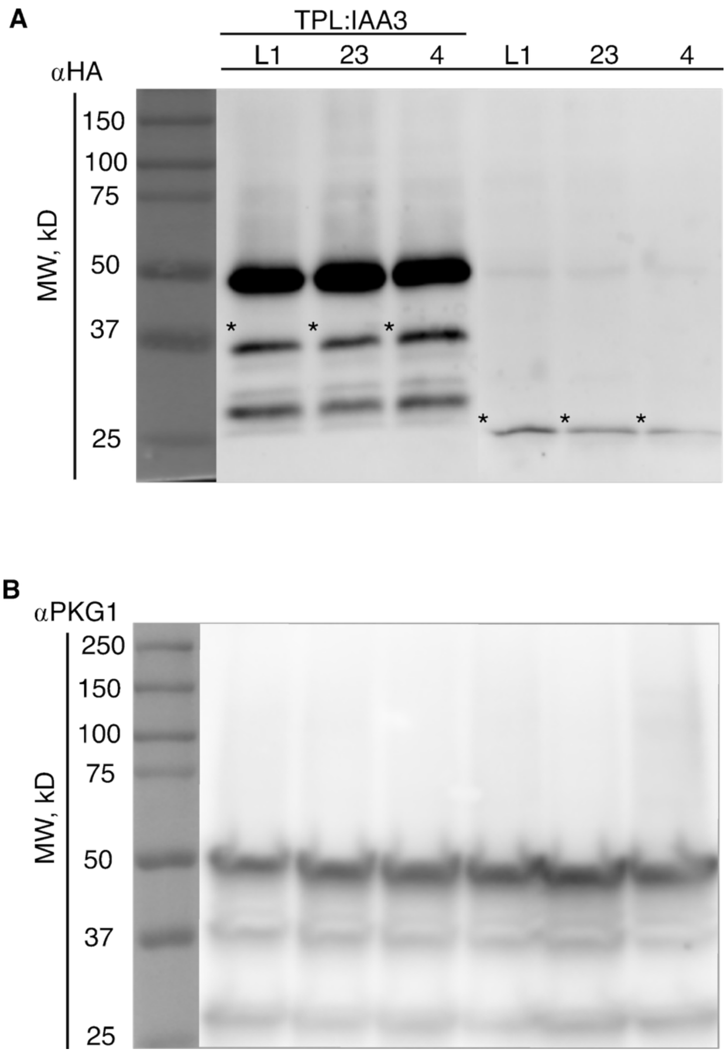

Figure 3 - figure supplement 2. TPL H8 EAR binding pocket cytometry protein expression.

(A) αHA Western blots of TPL H8 EAR subtype binding pocket cytometry stains blotting for TPL expression. Unfused or Free variants of L1, 23, and 4 primary band noted with asterisks (MW, KD = ~24) , Fused IAA3 variants primary band noted with asterisks (MW, KD = ~ 36). **(B)** αPkG1Western blots of TPL H8 EAR subtype binding pocket cytometry stains blotting for control yeast protein expression (MW, KD = ~45).

bioRxiv preprint doi: https://doi.org/10.64898/2026.06.02.729393; this version posted June 6, 2026. The copyright holder for this preprint (which was not certified by peer review) is the author/funder, who has granted bioRxiv a license to display the preprint in perpetuity. It is made available under a CC-BY 4.0 International license.

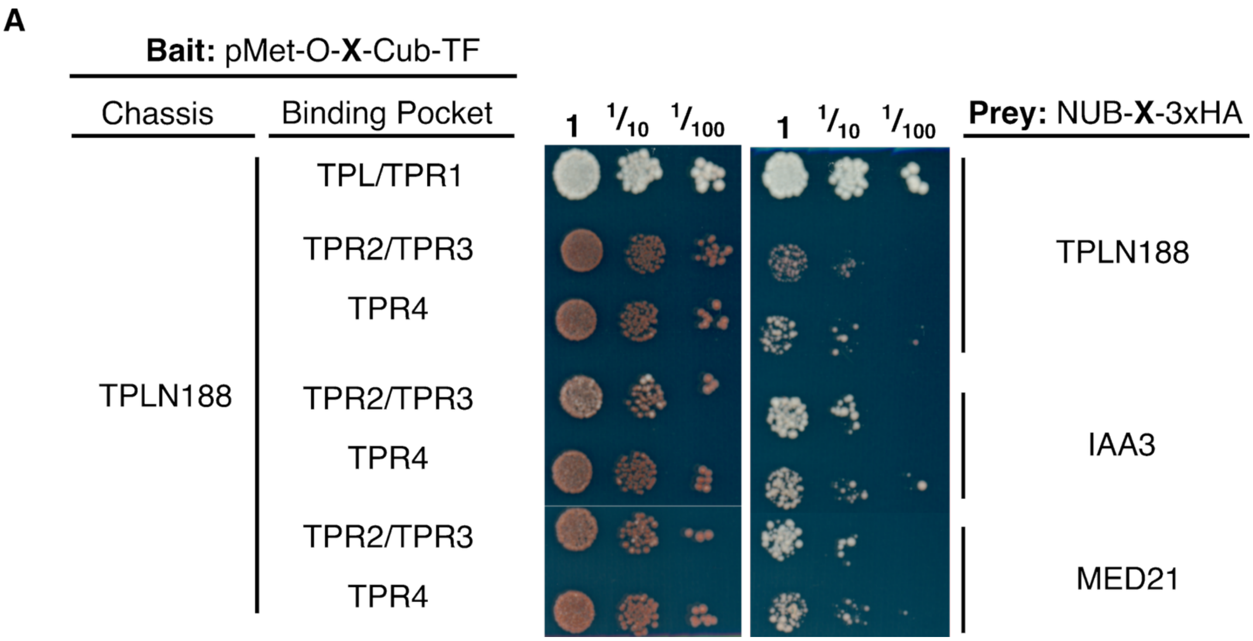

Figure 3 - figure supplement 3. TPX H8 variation effects on corepressor client binding.

(A) Growth control test (right) and interaction test (left) CytoSUS plates of H8 TPL variants (23 and 4) to test for physical interaction with known TPL interactors TPLN188, IAA3, and MED21

bioRxiv preprint doi: https://doi.org/10.64898/2026.06.02.729393; this version posted June 6, 2026. The copyright holder for this preprint (which was not certified by peer review) is the author/funder, who has granted bioRxiv a license to display the preprint in perpetuity. It is made available under a CC-BY 4.0 International license.

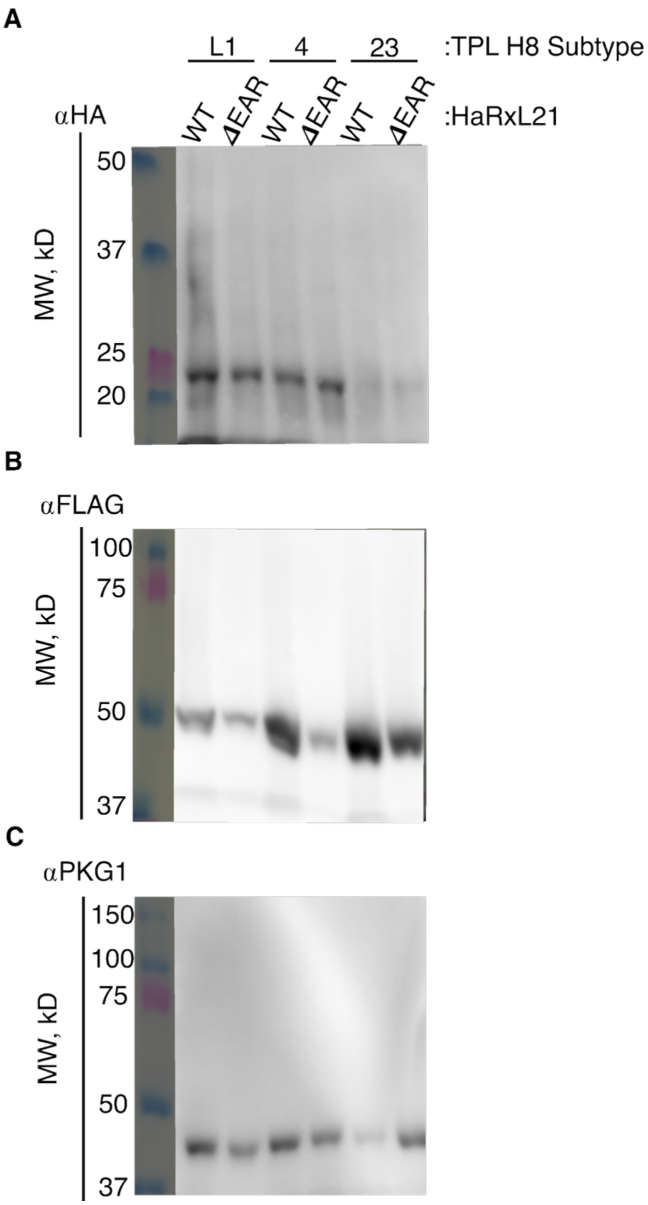

Figure 4 - figure supplement 1. TPX protein expression in RxL21 competition assays

(A) αHA Western blots of TPL H8 EAR subtype binding pocket with RxL21 (WT and *Δ*EAR) protein extracted from cytometry stains blotting for TPL expression. Subtypes L1, 23, and 4 primary band (MW, KD = ~24). **(B)** αFLAG Western blots of TPL H8 EAR subtype binding pocket with RxL21 (WT and *Δ*EAR) cytometry stains blotting for RxL21 (WT or *Δ*EAR) protein expression. RxL21 Wt and *Δ*EAR primary band (MW, KD = ~49). **(C)** αPkG1Western blots of TPL H8 EAR subtype binding pocket with RxL21 (WT and *Δ*EAR) cytometry stains blotting for control yeast protein expression (MW, KD = ~45).

bioRxiv preprint doi: https://doi.org/10.64898/2026.06.02.729393; this version posted June 6, 2026. The copyright holder for this preprint (which was not certified by peer review) is the author/funder, who has granted bioRxiv a license to display the preprint in perpetuity. It is made available under a CC-BY 4.0 International license.

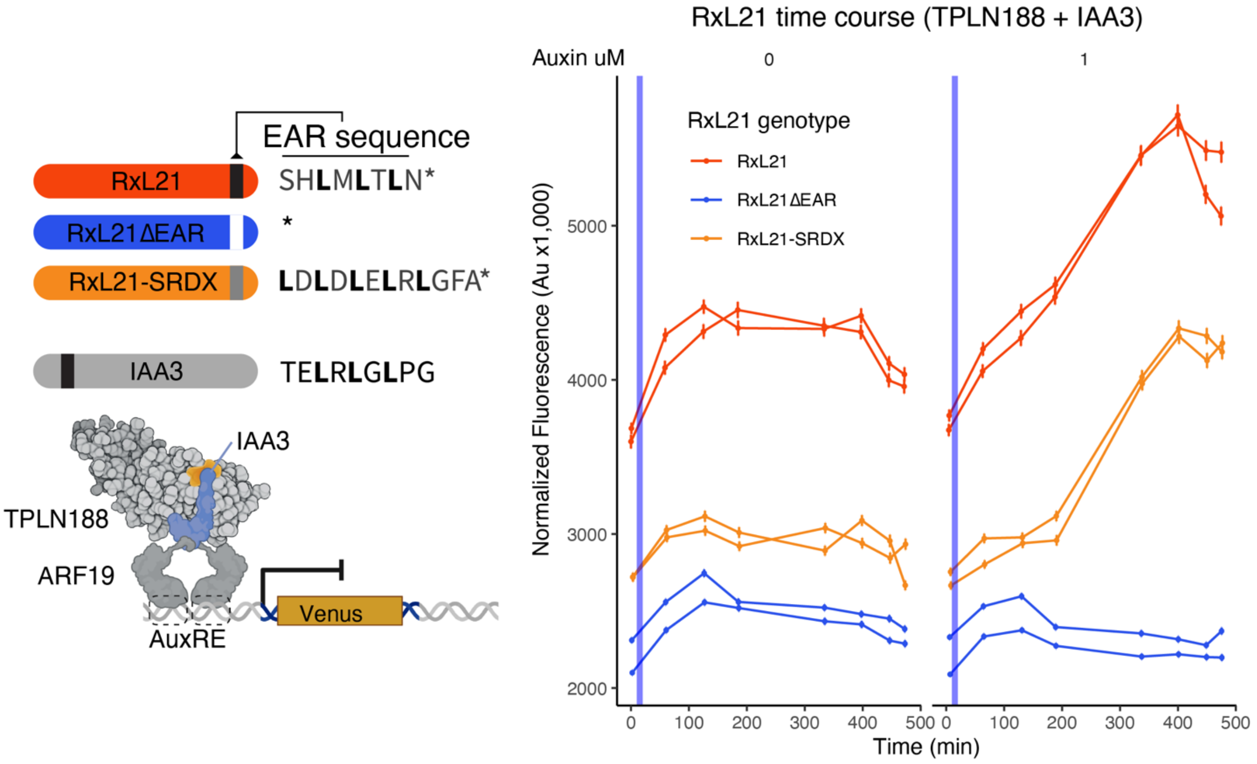

Figure 4 - figure supplement 2. Auxin effects on RxL21 competed TPX repression Time course flow cytometry of previously tested RxL21 competition strains with TPLN188 (Figure 5B) with the addition of Auxin (vertical blue bar) to further sensitize the system to RxL21 disruption by promoting the degradation of IAA3. Wild type RxL21 (Red), RxL21 with deletion of the C-terminal EAR motif (blue), and replacement of the native EAR sequence with the SRDX EAR sequence (orange) strains were treated with either no auxin (mock, left panel), or 1μM IAA (right panel), and cytometry was performed over the following 500 minutes. Every point represents the average fluorescence of 5–10,000 individually measured yeast cells (a.u.: arbitrary units). Auxin (IAA-10 μM) was added at the indicated time (gray bar, +Aux). Error bars represent standard error.

bioRxiv preprint doi: https://doi.org/10.64898/2026.06.02.729393; this version posted June 6, 2026. The copyright holder for this preprint (which was not certified by peer review) is the author/funder, who has granted bioRxiv a license to display the preprint in perpetuity. It is made available under a CC-BY 4.0 International license.

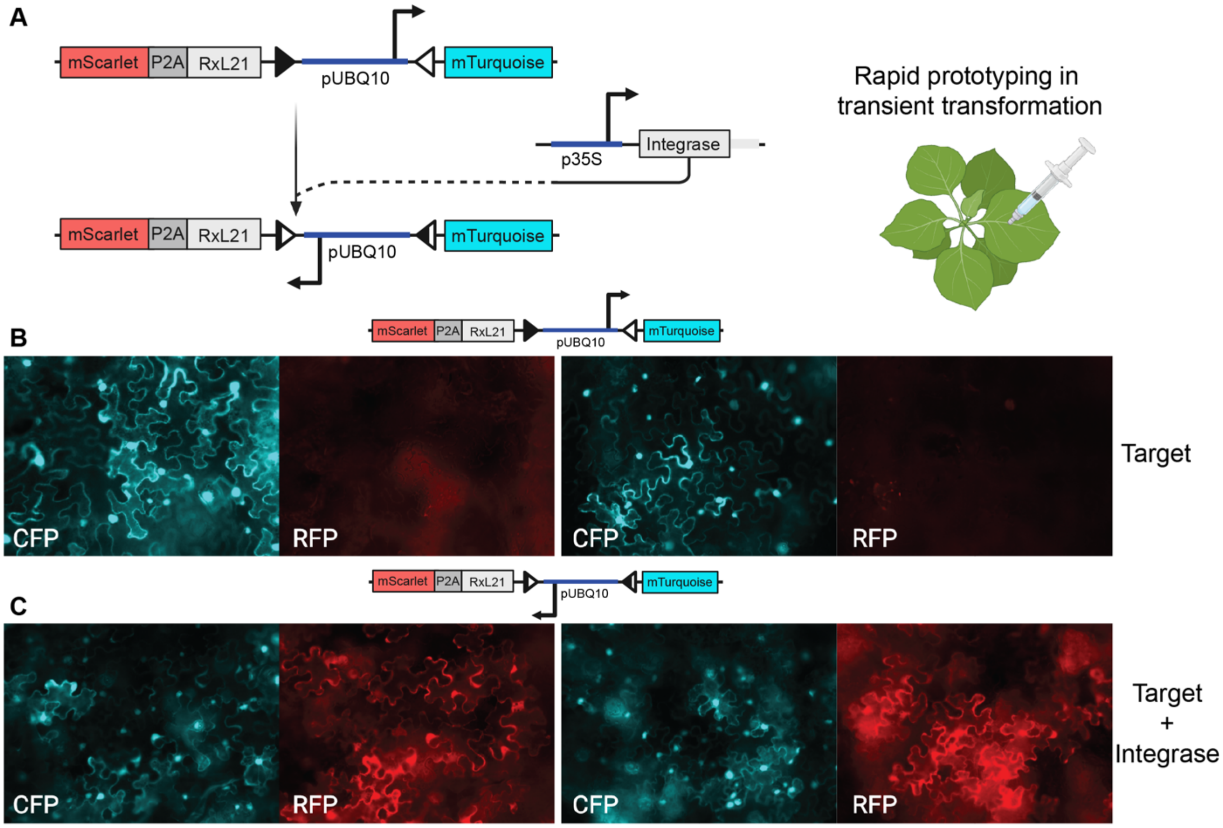

Figure 4 - figure supplement 3. Rapid prototyping of iInducer constructs in transient transfections of *Nicotiana benthamiana* at 2 days after injection. (A) Design of the integrase target. The target is composed of two PhiC31 integrase sites (triangles) surrounding a constitutive promoter (pUBQ10), the fluorescent reporter mTurquoise and the RxL21 CDS linked to a P2A cleavage sequence and a fluorescent reporter (mScarlet). In the absence of integrase mTurquoise is expressed. In the presence of integrase, the integrase (PhiC31) mediates inversion of the DNA between the integrase sites, inverting the promoter and leading to RxL21 and mScarlet expression. The expression of the integrase is mediated by the selected promoter selected, here the viral promoter p32S. **(B)** On the left side is the wild-type RxL21-P2A-mScarlet target that switches from mTurquoise to RxL21-P2A-mScarlet alone (top) and **(C)** with a *p35S:PhiC31* construct (bottom). **(B)** On the right side shows the RxL21ΔEAR-P2A-mScarlet target alone (top) and (**C**) RxL21ΔEAR-P2A-mScarlet target with a *p35S:PhiC31* construct (bottom). Microscopy images were taken on a 20x objective to allow a wide view of switching efficiency.

bioRxiv preprint doi: https://doi.org/10.64898/2026.06.02.729393; this version posted June 6, 2026. The copyright holder for this preprint (which was not certified by peer review) is the author/funder, who has granted bioRxiv a license to display the preprint in perpetuity. It is made available under a CC-BY 4.0 International license.

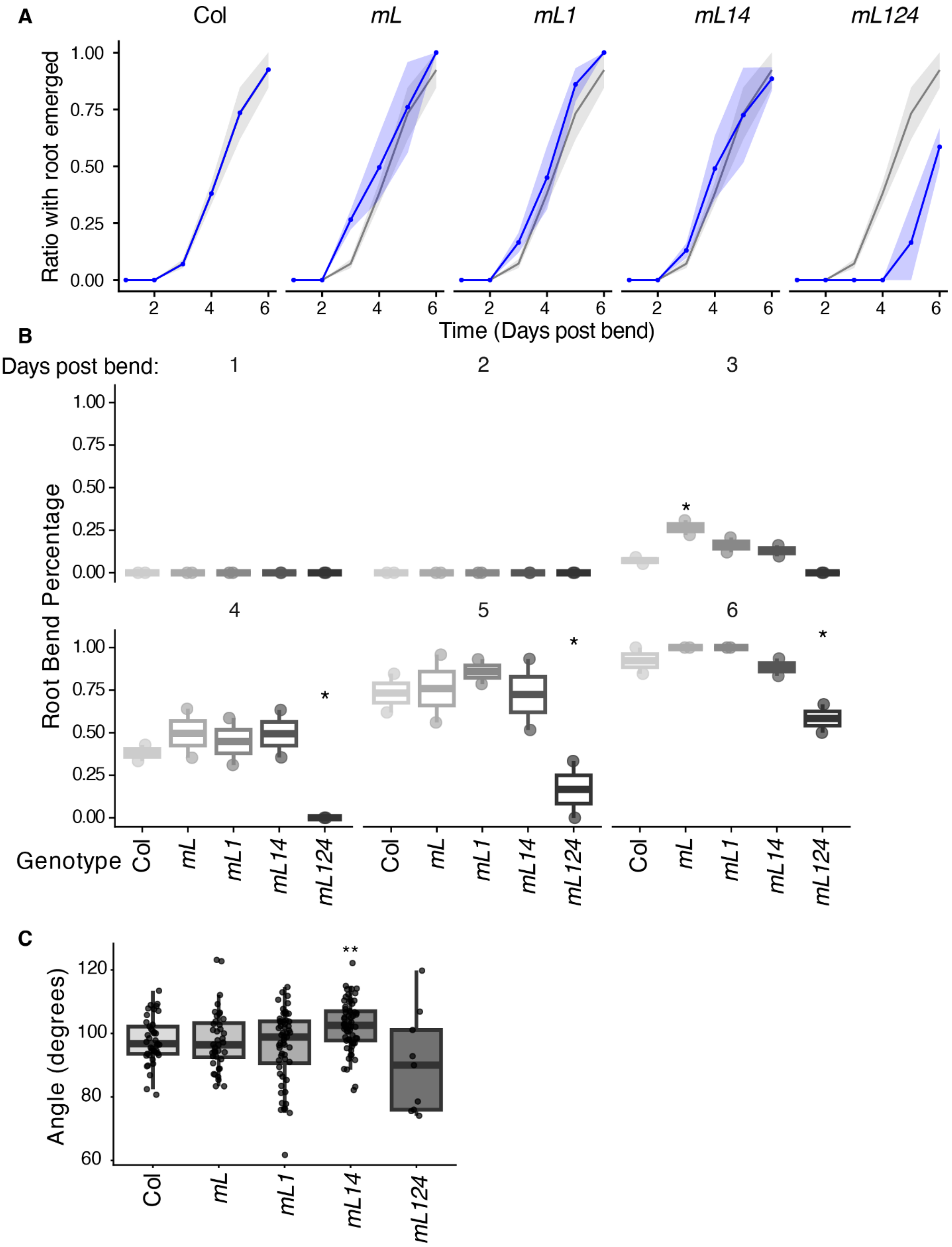

Figure 5 - figure supplement 1. Root phenotypes in TPX multiple mutants.

bioRxiv preprint doi: https://doi.org/10.64898/2026.06.02.729393; this version posted June 6, 2026. The copyright holder for this preprint (which was not certified by peer review) is the author/funder, who has granted bioRxiv a license to display the preprint in perpetuity. It is made available under a CC-BY 4.0 International license.

(A-B) Time course of lateral root emergence at the bend site. Mean proportion of seedlings for Col, *tpr1 (m1)*, *tpl tpr1 (mL1)*, *tpl tpr1 tpr4 (mL14)*, and *tpl tpr1 tpr2 tpr4 (mL124)* genotypes across 1–6 days post-bend (dpb), with an emerged lateral root at the bend site plotted over 1–6 days post-bend (dpb) for each genotype. **(A).** Each panel shows an individual genotype (blue line and shaded ribbon, indicating the range across 2 plates) overlaid on the Wild-type (Col-0) reference (grey line and shaded ribbon). Seedlings were rotated 90° to induce gravitropic bending and scored daily. n = 2 plates per genotype per timepoint. **(B)** Box plot - boxes represent the interquartile range, with the median shown as a horizontal line; individual plate values are overlaid as points. Significance relative to Col was assessed at each day using Fisher's exact test on pooled counts across plates, with Benjamini-Hochberg correction for multiple comparisons. * $p < 0.05$. n = 2 plates per genotype per timepoint. **(C)** Root bend angles across genotypes. Angles of the primary root at the bend site measured from individual seedlings at 2 days post-bend for Col, *tpr1 (m1)*, *tpl tpr1 (mL1)*, *tpl tpr1 tpr4 (mL14)*, and *tpl tpr1 tpr2 tpr4 (mL124)* genotypes. Boxes represent the interquartile range with the median shown as a horizontal line; individual root measurements are overlaid as points. Angles were measured from TIFF images using ImageJ. Significance relative to Col was assessed using a Wilcoxon rank-sum test with Benjamini-Hochberg correction for multiple comparisons. ** $p < 0.01$. n = Col: 41, *tpr1*: 40, *tpl tpr1*: 56, *tpl tpr1 tpr4*: 62, *tpl tpr1 tpr4 tpr2*: 9 roots across 2 plates.

bioRxiv preprint doi: https://doi.org/10.64898/2026.06.02.729393; this version posted June 6, 2026. The copyright holder for this preprint (which was not certified by peer review) is the author/funder, who has granted bioRxiv a license to display the preprint in perpetuity. It is made available under a CC-BY 4.0 International license.

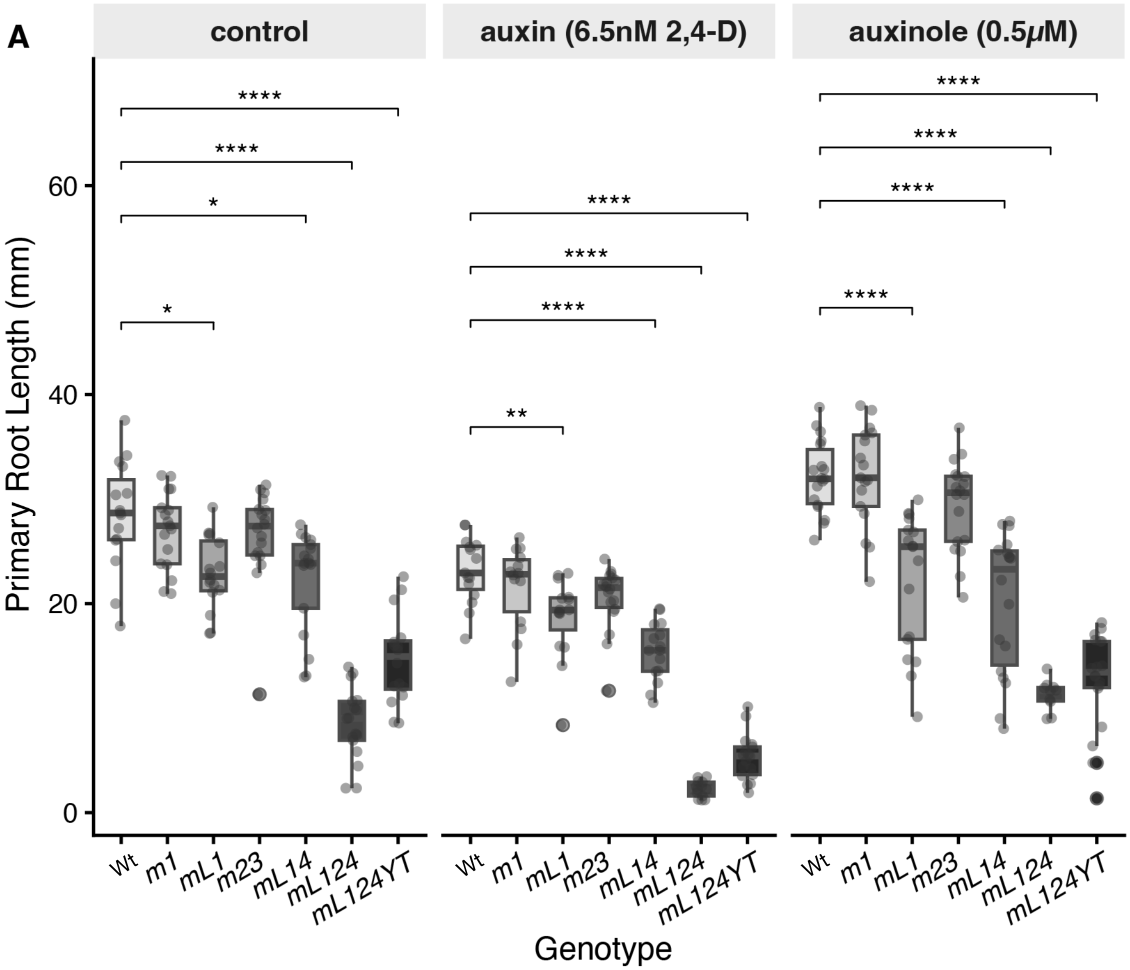

Figure 5 - figure supplement 2. Primary root length of *Arabidopsis thaliana* TPX mutants in response to auxin and auxinole. 9A-B) Primary root length of wild-type (Col-0) and TPX mutants Col-0 (WT), *tpr1* (*m1*), *tpl tpr1* (*mL1), tpr2 tpr3* (*m23*), *tpl tpr1 tpr4* (*mL14*), *tpl tpr1 tpr2 tpr4* (*mL124*) and *tpl tpr1 tpr2 tpr4 YFP:TPL* (*mL124YL*) seedlings grown vertically on ½ LS medium supplemented with 6.5 nM 2,4-D (auxin) or 0.5 μM auxinole for 4 days. Seedlings were photographed and root length measured using Fiji. Boxplots show the median (center line), interquartile range (box), and 1.5× IQR (whiskers); individual measurements are overlaid as points. Statistical comparisons between each genotype and Col-0 within each treatment were performed using a two-tailed Student's t-test with Bonferroni correction. Significance is indicated as: * $p \leq 0.05$, ** $p \leq 0.01$, *** $p \leq 0.001$, **** $p \leq 0.0001$; ns, not significant. n = 18 seedlings per genotype per treatment pooled from 3 independent experiments.

bioRxiv preprint doi: https://doi.org/10.64898/2026.06.02.729393; this version posted June 6, 2026. The copyright holder for this preprint (which was not certified by peer review) is the author/funder, who has granted bioRxiv a license to display the preprint in perpetuity. It is made available under a CC-BY 4.0 International license.

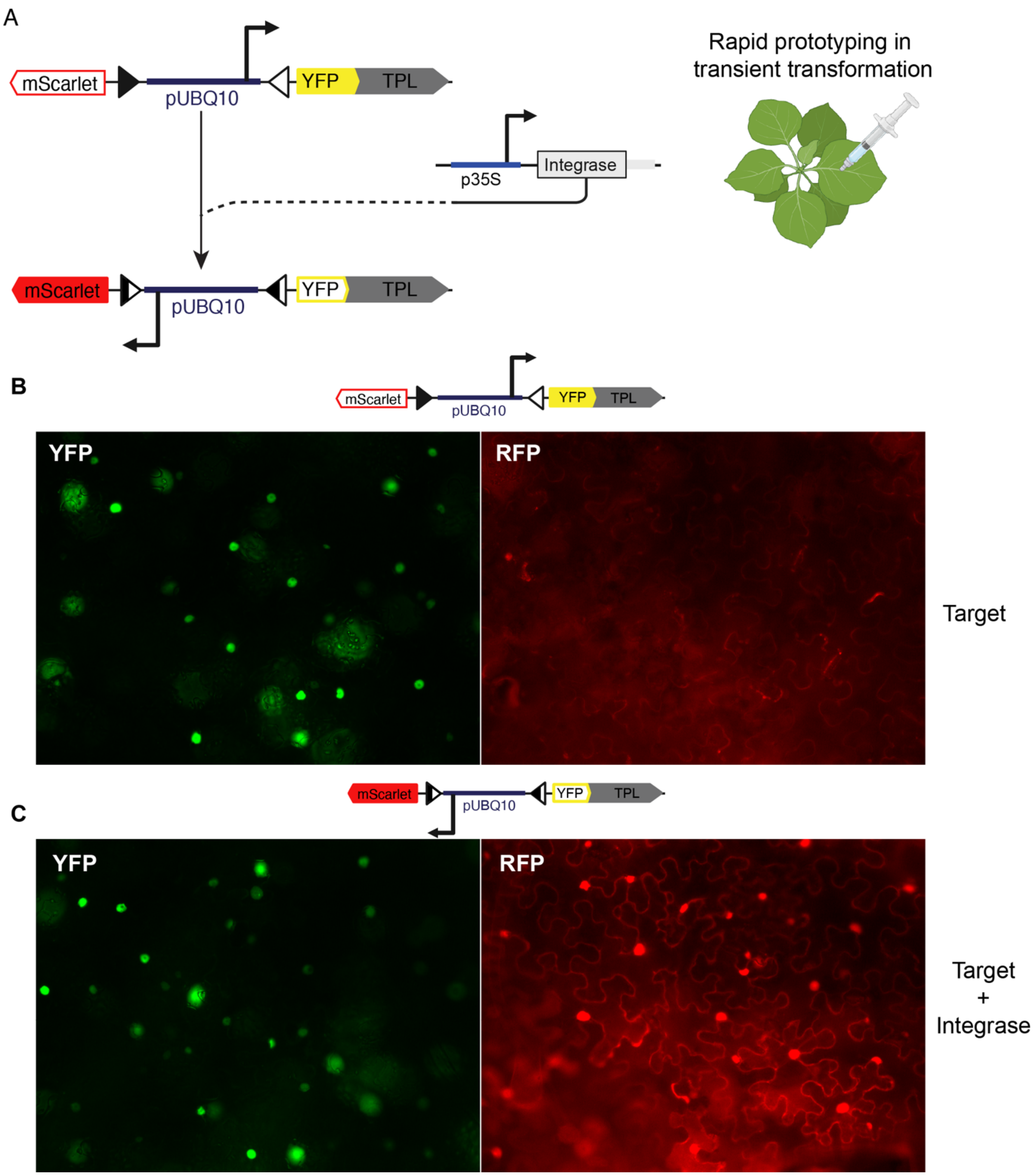

Figure 5 - figure supplement 3. Rapid prototyping of iEraser constructs in transient transfections of *Nicotiana benthamiana* at 2 days after injection. (A) Design of the integrase target. The target is composed of two PhiC31 integrase sites (triangles) surrounding a constitutive promoter (pUBQ10), the YFP-tagged TPL full length CDS and a fluorescent reporter (mScarlet). In the absence of integrase YFP-TPL is expressed. In the presence of integrase, the integrase (PhiC31) mediates inversion of

bioRxiv preprint doi: https://doi.org/10.64898/2026.06.02.729393; this version posted June 6, 2026. The copyright holder for this preprint (which was not certified by peer review) is the author/funder, who has granted bioRxiv a license to display the preprint in perpetuity. It is made available under a CC-BY 4.0 International license.

the DNA between the integrase sites, inverting the promoter and leading to mScarlet expression. The expression of the integrase is mediated by the selected promoter selected, here the viral promoter p32S. **(B)** iEraser target expression in the absence of integrase. **(C)** iEraser target expression in the presence of a *p35S:PhiC31* construct. On the left side. On the left side is the YFP channel for YFP-TPL, and mScarlet is on the right side. Microscopy images were taken on a 20x objective to allow a wide view of switching efficiency.

bioRxiv preprint doi: https://doi.org/10.64898/2026.06.02.729393; this version posted June 6, 2026. The copyright holder for this preprint (which was not certified by peer review) is the author/funder, who has granted bioRxiv a license to display the preprint in perpetuity. It is made available under a CC-BY 4.0 International license.

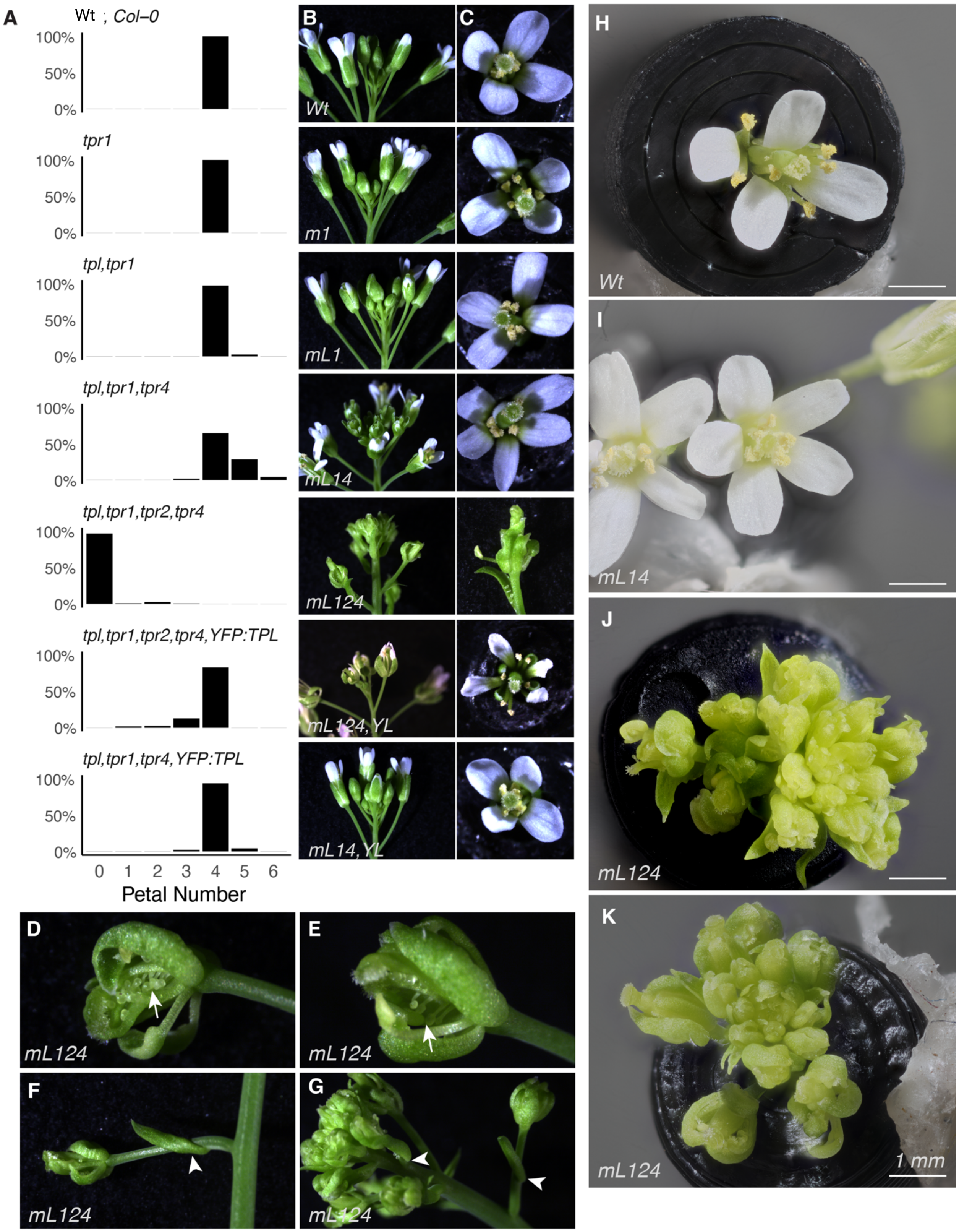

Figure 5 - figure supplement 4. Flower phenotypes of TPX mutants. (A) Flower petal quantification of 150 flowers for each genotype data is presented as the ratio of

bioRxiv preprint doi: https://doi.org/10.64898/2026.06.02.729393; this version posted June 6, 2026. The copyright holder for this preprint (which was not certified by peer review) is the author/funder, who has granted bioRxiv a license to display the preprint in perpetuity. It is made available under a CC-BY 4.0 International license.

the total. Wild-type (Col-0) and TPX mutants Col-0 (WT), *tpr1* (*m1*), *tpl tpr1* (*mL1), tpr2 tpr3* (*m23*), *tpl tpr1 tpr4* (*mL14*), *tpl tpr1 tpr2 tpr4* (*mL124*) and *tpl tpr1 tpr2 tpr4 YFP:TPL* (*mL124YL*) **(B)** Column is inflorescence **(C)** Column is individual flowers from above **(D-E)** Highlighting ectopic ovule formation (arrow). **(F-G)** Highlight novel induction of bracts (arrow). **(H-K)** MacroPod PRO images for high resolution focus stacked observation of floral morphology in Wt, *mL14*, and *mL124*.

bioRxiv preprint doi: https://doi.org/10.64898/2026.06.02.729393; this version posted June 6, 2026. The copyright holder for this preprint (which was not certified by peer review) is the author/funder, who has granted bioRxiv a license to display the preprint in perpetuity. It is made available under a CC-BY 4.0 International license.

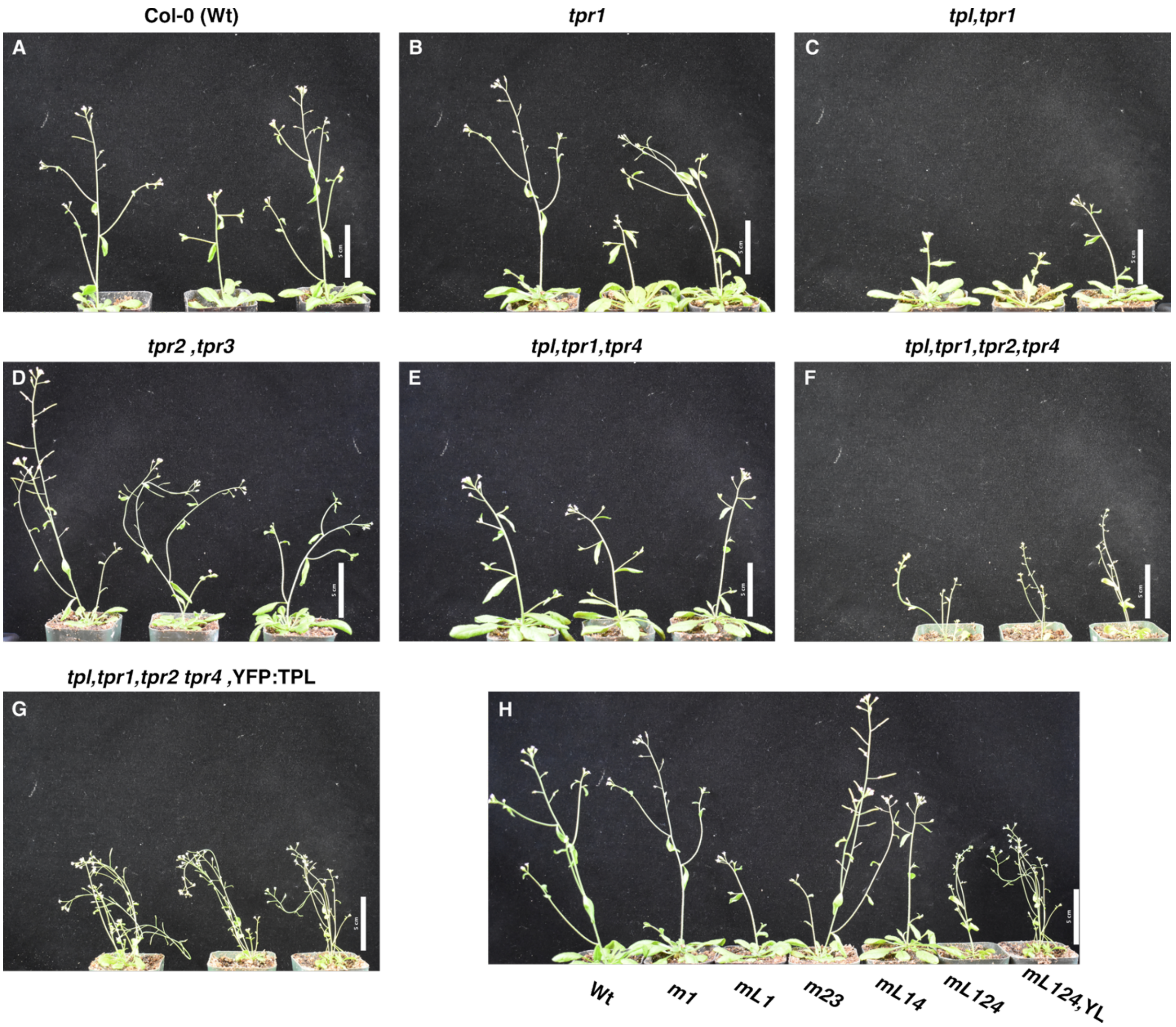

Figure 5 - figure supplement 5. Whole plant phenotypes of TPX mutants.
(A) Three whole plant representatives of wild-type Col-0 (Wt) genotype with a 5 cm. scale bar. **(B)** Three whole plant representatives of *tpr1* (*m1)* genotype with a 5 cm. scale bar. **(C)** Three whole plant representatives of *tpl tpr1* (*mL1)* genotype with a 5 cm. scale bar. **(D)** Three whole plant representatives of *tpr2 tpr3* (*m23*) genotype with a 5 cm. scale bar. **(E)** Three whole plant representatives of *tpl tpr1 tpr4* (*mL14*) genotype with a 5 cm. scale bar. **(F)** Three whole plant representatives of *tpl tpr1 tpr2 tpr4* (*mL124*) genotype with a 5 cm. scale bar. **(G)** Three whole plant representatives of *tpl tpr1 tpr2 tpr4 YFP:TPL* (*mL124,YL*) genotype with a 5 cm. scale bar. **(H)** Full plant images of representative member of each genotype Wt and TPX mutants: *m1*, *mL1, m23*, *mL14*, *mL124*, and *mL124,YL* with a 5 cm. scale bar.
